# Enzymatic Preparation of Low-Molecular-Weight *Laminaria japonica* Polysaccharides and Evaluation of Its Effect on Modulating Intestinal Microbiota in High-Fat-Diet-Fed Mice

**DOI:** 10.3389/fbioe.2021.820892

**Published:** 2022-02-14

**Authors:** Xiaodan Fu, Yuming Zhan, Nannan Li, Dongxing Yu, Wei Gao, Ziqiang Gu, Lin Zhu, Rong Li, Changliang Zhu

**Affiliations:** ^1^ State Key Laboratory of Food Science and Technology, China-Canada Joint Laboratory of Food Science and Technology (Nanchang), Key Laboratory of Bioactive Polysaccharides of Jiangxi Province, Nanchang University, Nanchang, China; ^2^ Shandong Feed and Veterinary Drug Quality Center, Jinan, China; ^3^ College of Food Science and Engineering, Ocean University of China, Qingdao, China; ^4^ SOHAO FD-TECH CO., LTD., Qingdao, China; ^5^ Qingdao Women and Children’s Hospital, Qingdao, China

**Keywords:** *Laminaria japonica*, cellulase, alginate lyase, low-molecular-weight polysaccharides, high-fat diet, intestinal microbiota

## Abstract

Recent studies have shown that seaweed polysaccharides can ameliorate high-fat-diet (HFD)-induced metabolic syndromes associated with the regulatory function of gut microbiota. However, kelp, a natural source of seaweed polysaccharides, is highly viscous, making it difficult to prepare dietary fiber by simple degradation. Therefore, we developed a novel method of preparing low-molecular-weight polysaccharides from *Laminaria japonica* by combining high-pressure pretreatment and composite enzymatic degradation and evaluated the obesity prevention activity of these polysaccharides. Seaweed *L. japonica* polysaccharides (SJP) were rapidly utilized by the human fecal microbiota *in vitro*, resulting in the generation of short-chain fatty acids (SCFAs), specifically acetate and propionate. The *in vivo* effects of SJP on the intestinal microbiota were also investigated using HFD-fed C57BL/6J mice. SJP reduced weight gain and fat deposition in HFD-fed mice and increased the concentration of total SCFAs, including acetate, propionate, and butyrate in the feces. SJP ameliorated HFD-induced gut microbiota dysbiosis, resulting in increased abundance of *Faecalibaculum*, *Romboutsia*, and *Clostridium sensu stricto 1* and decreased abundance of *Blautia* and *Lactobacillus*. Further, SJP enhanced the abundance of *Akkermansia muciniphila* in mice provided with HFD and normal chow. Single-strain culture experiments also revealed that SJP promoted the growth of *A. muciniphila*. This study highlights the potential use of SJP, prepared using composite enzymatic degradation (cellulase and recombinant alginate lyase), in preventing obesity and restoring intestinal homeostasis in obese individuals.

## 1 Introduction

Obesity is a metabolic disorder characterized by excessive accumulation of fat, increased waist circumference, and excessive body mass index, which is related to type 2 diabetes and cardiovascular disease (Gérard, 2016). Further, obesity has been regarded as a major public health threat. Strategies, such as weight loss drugs, reduced caloric intake, and increased exercise, are typically employed for obesity prevention. However, no available treatment has shown long-term success, and this has been attributed to complex individual differences and changes in environmental factors that have limited the effectiveness of obesity treatments (Blüher, 2019). The only commercially available medication for the long-term treatment of obesity is orlistat; however, its use is known to be associated with various unpleasant gastrointestinal side effects (Topaloglu and Sahin, 2021). Hence, the development and use of anti-obesity agents from natural materials are important.

A growing number of studies have shown that the intestinal microbiota plays a significant role in the development of obesity and associated metabolic comorbidities (Haslam et al., 2006; Gérard, 2016). Dietary factors play a prominent role in altering the diversity and composition of the intestinal microbiota. For example, they specifically promote the growth of beneficial intestinal species that produce high levels of bioactive metabolites (Kasubuchi et al., 2015). Nowadays, seaweeds have attracted much attention for their rich diversified structures, various biological activities, and promising further application as functional foods; in particular, it is known to improve intestinal function (Li et al., 2020; Otero et al., 2021). Recent studies have shown that seaweed polysaccharides can ameliorate high-fat diet (HFD)-induced metabolic syndromes by promoting the proliferation of beneficial intestinal microorganisms, for example, *Akkermansia* (Shang et al., 2017; Guo et al., 2019; Wang et al., 2020). However, in comparison with terrestrial neutral polysaccharides, seaweed polysaccharides are usually utilized slowly by lactic acid bacteria and Bifidobacteria. Thus, it is necessary to perform more systematic analyses from the perspective of the structure of intestinal microbiota.

*Laminaria japonica*, one of the widely used edible algae, has been reported for its multiple physiological functions including antioxidant, anti-inflammatory, and hypoglycemic and hypolipidemic effects (Li et al., 2021). Polysaccharides, mostly found as part of cellular walls, have been reported to be the major bioactive compounds in *L. japonica* to achieve biological functions. However, they are highly viscous, making them difficult to prepare using simple extraction and degradation. Thus, environmentally friendly preparation technologies with both high yield and quality are necessary for obtaining rich extracts (Dobrinčić et al., 2020; Otero et al., 2021). Therefore, we established a novel method using high-pressure pretreatment combined with composite enzyme degradation to prepare low-molecular-weight (low-MW) seaweed *L. japonica* polysaccharides (SJP) using cellulase and recombinant alginate lyase. Cellulase is capable of degrading the cellulose located in the seaweed cell wall to enhance the extraction efficiency of water-soluble polysaccharides. Subsequently, the recombinant alginate lyase is assumed to hydrolyze alginate and to obtain low-MW polysaccharides. To evaluate the potential anti-obesity and modulation effect on intestinal microbiota, SJP were exposed to human fecal microbiota *in vitro* and were also used as a supplement for HFD-fed mice. The present study may represent a potentially simple and effective approach for preparing low-MW seaweed polysaccharides for further commercial application.

## 2 Materials and Methods

### 2.1 Materials

Dried *L. japonica* was purchased from an aquatic market in Rongcheng, China. Cellulase (5,000 U/g) was purchased from Sunson Industry Group Co., Ltd. (Beijing, China). Recombinant alginate lyase 102C300C, provided by Applied Microbiology Laboratory (Ocean University of China, Qingdao, China), was used for the preparation of alginate oligosaccharides (Algo) (Yang et al., 2020). The 102C300C was endotype, which could degrade both guluronate and mannuronate, but had a preferential polyG substrate specificity (Yang et al., 2018; Yang et al., 2020). Briefly, the enzyme activity of 102C300C was 249.6 U/mg when alginate was used as the substrate. Alginate was degraded by 102C300C at 150 U/g in 100 mM of phosphate buffer (pH 6.0). The hydrolysis reaction was conducted at 50°C for 6 h and was terminated by heating to 90°C for 10 min. The supernatant was obtained after centrifugation at 10,000 × *g* at 4°C for 10 min. The high-MW fraction was removed by a 3-fold ethanol precipitation. The supernatant was then subjected to a 5-fold ethanol precipitation, and the precipitate was collected for subsequent experimental analysis. The obtained Algo precipitate exhibited an MW of below 2.5 kDa and a β-d-mannuronate/α-l-guluronate ratio of 0.37. Acetic, propionic, *n*-butyric, i-butyric, *n*-valeric, and i-valeric acids were purchased from Sigma-Aldrich (Bellefonte, PA, USA). Formic acid (Tokyo Chemical Industry Co., Ltd., Tokyo, Japan) and lactic acid (Dr. Ehrenstorfer GmbH, Augsburg, Germany) were also used in this study. Mannose, glucose, galactose, glucuronic acid, xylose, rhamnose, arabinose, ribose, fucose (Sigma-Aldrich, St Louis, MO, USA), guluronic acid, and mannuronic acid (Qingdao Bozhi Huili Biotechnology Co., Ltd., Qingdao, China) were used as standards. *Akkermansia muciniphila* DSM22959 was purchased from the Leibniz Institute DSMZ-German Collection of Microorganisms and Cell Cultures (Braunschweig, Germany). All chemicals and reagents were of analytical grade.

### 2.2 Preparation of Seaweed *Laminaria japonica* Polysaccharides

Dried *L. japonica* was washed with distilled water to remove salt, further dried using hot air at 55°C for 48 h, ground using a DFY-400 crusher (Wenling Linda Machinery Co., Ltd., Zhejiang, China), and sieved using an 80-mesh sieve to obtain a uniform powder. The powder was mixed with distilled water at 30% (w/v), pressure pretreated at 0.2 MPa for 10 min (DN900-1800-6, Zhonglian Puhui Intelligent Equipment Co., Ltd., Shandong, China), and enzymatically degraded using 150 U/g of recombinant alginate lyase 102C300C and 200 U/g of cellulase at 50°C in 100 mM of phosphate buffer (pH 6.0) for 6 h in a shaking water bath. The hydrolysate was heated at 90°C for 10 min to terminate the reaction. The supernatant was collected by centrifugation at 10,000 × *g* for 10 min, concentrated to 1/4 of the initial volume, and precipitated using 5-fold (v/v) ethanol at 4°C for 24 h. After centrifugation, the precipitate was obtained; proteins were removed using 3 rounds of 4:1 (v/v) CHCl_3_-*n*-BuOH treatment, as per the Sevag method (Zha et al., 2012). Finally, the SJP sample was dialyzed using biotech-grade CE dialysis tubing with an MW cutoff of 500 Da (Repligen Spectrum™ 131060, Waltham, MA, USA) against distilled water for 40 h for the removal of salt and monosaccharide and further freeze-dried. Under the same technological condition, the pressure-pretreated powder was respectively degraded using single alginate lyase 102C300C (SJP-A) or cellulase (SJP-C) for comparison.

The extraction yield was calculated using the following equation:

$$extraction\;yield\;\left(\%\right)=\frac{A}{B}\times 100$$

where A is the weight of dried SJP, SJP-A, and SJP-C, respectively; and B is the weight of dried *L. japonica* powder.

### 2.3 General Analysis of Seaweed *Laminaria japonica* Polysaccharides

The protein, moisture, and ash content of the SJP, SJP-A, and SJP-C sample were measured in accordance with the AOAC standard methods (AOAC International, 2005). The total carbohydrate content was determined using the phenol–sulfuric acid method (Dubois et al., 1956). The uronic acid and sulfate group contents were determined using the *m*-hydroxydiphenyl (Blumenkrantz and Asboe-Hansen, 1973) and barium sulfate turbidimetric methods (Dodgson, 1961), respectively. The monosaccharide component was analyzed on an Agilent 1260 Infinity high-performance liquid chromatography (HPLC) system coupled to a ZORBAX Eclipse XDB-C18 column (4.6 mm × 150 mm, 5 µm; Agilent Technologies, Santa Clara, CA, USA). Samples were acid-hydrolyzed using 2 mol/L of trifluoroacetic acid at 110°C for 6 h. Further derivatization and HPLC evaluation were conducted in accordance with methods proposed in a previous study (Fu et al., 2019). The MWs were measured using a TSKgel G4000PW_XL_ column (7.8 mm × 300 mm × 10 μm, Tosoh Co., Ltd., Japan) equipped with a refractive index detector (G1362A; Agilent Technologies). Dextran standards of 1, 3.65, 5, 12, 21, and 80 kDa were used for calibration. The following experimental parameters were used: column temperature, 35°C; flow rate, 0.3 ml/min; and mobile phase, 10 mM of NaH_2_PO_4_ and 200 mM of NaNO_3_ dissolved in ultrapure water.

### 2.4 *In Vitro* Treatment of Seaweed *Laminaria japonica* Polysaccharides Using Human Feces

#### 2.4.1 *In Vitro* Treatment

Human fecal samples were obtained from three healthy adult volunteers (22–25 years old, two women and one man). The present study was approved by the ethics committee of Qingdao Women and Children’s Hospital (Qingdao, China) (approval no. QFELL-KY-2019-68) in accordance with the standards of the Declaration of Helsinki and current ethical guidelines. The volunteers had not been treated with antibiotics or other medications for at least 3 months prior to feces collection and had no history of gastrointestinal disorders. Fresh fecal samples were diluted with 0.01 M of phosphate-buffered saline (pH 7.0, containing 0.5 g/L of l-cysteine hydrochloride) to obtain a 20% (w/v) fecal slurry. The fecal slurries were filtered using four layers of filter cloth and stored in sealed bottles at 37°C for further inoculation. Media were prepared based on a previously reported method (Fu et al., 2020). Samples of 0.6 g of SJP, Algo, fucose, and glucose were each dissolved in 54 ml of fermentation medium in 100-ml serum bottles and aerated with a mixture of H_2_ (10%), CO_2_ (10%), and N_2_ (80%) for 15 min, followed by sealing using butyl rubber stoppers and aluminum crimp seals. A 6-ml fecal slurry was inoculated with 54 ml of each dissolved polysaccharide after preheating the medium at 37°C. A control group was prepared by omitting the inoculation step. All samples were cultured in a YQX-T anaerobic incubator (Longyue Instrument Equipment Co., Ltd., Shanghai, China). Reaction products were collected at 0, 6, 12, 24, and 48 h, followed by centrifugation at 10,000 × *g* for 5 min at 4°C. The supernatant was retained for further analysis.

#### 2.4.2 Profile of Carbohydrate Utilization

The carbohydrate utilization of SJP was characterized using a TSKgel G4000PW_XL_ column according to the procedure described in Section 2.3. To obtain a more detailed analysis, the carbohydrate utilization of SJP and Algo was also profiled using thin-layer chromatography (TLC), as described previously (Su et al., 2017). The major monosaccharides in SJP including glucose, mannuronic acid, guluronic acid, and fucose and the origin alginate sample were used for reference.

#### 2.4.3 Determination of pH Variation and Short-Chain Fatty Acid Production

pH variation was measured using LAQUAtwin-pH-22 (Horiba Stec Co., Ltd., Tokyo, Japan). Short-chain fatty acid (SCFA) production was determined using a method published in our previous paper (Fu et al., 2020). The experimental parameters used were as follows: mobile phase, 1% H_3_PO_4_; column temperature, 60°C; and flow rate, 0.85 ml/min. Formic acid (15.83 mmol/L), lactic acid (10.96 mmol/L), acetic acid (17.25 mmol/L), propionic acid (13.24 mmol/L), i-butyric acid (10.65 mmol/L), *n*-butyric acid (10.76 mmol/L), and i-valeric acid (8.94 mmol/L) were used as experimental standards, and *n*-valeric acid (9.08 mmol/L) was used as an internal standard. A chromatogram of the experimental standards is shown in Supplementary Figure S1 of the Supplementary Material.

### 2.5 Animals and Diets

The effect of SJP on the intestinal microbiota was investigated in mice fed an HFD. Animal experiments were performed in accordance with the guidelines proposed by the ethical committee of the College of Food Science and Engineering, Ocean University of China (Qingdao, China) (approval no. SPXY2018039). Thirty male C57BL/6J mice (5 weeks old; specific pathogen-free) were obtained from Beijing Vital River Laboratory Animal Technology Co., Ltd. (Beijing, China). All the mice were housed in a stable environment (12-h light/dark cycle, 23–26°C) and adaptively fed for 2 weeks, with free access to water and a normal chow diet (Beijing Keaoxieli Co., Ltd., Beijing, China). After acclimation, mice were randomly and evenly divided into the following five groups: 1) the HFD group, mice were fed HFD (5.24 kcal/g, D12492, 60% kcal from fat, Research Diets); 2) the HSJP group, mice were fed HFD and treated with 2 g/kg of SJP; 3) the HO group, mice were fed HFD and treated with 60 mg/kg of orlistat (positive control); 4) the NCD group, mice were fed a normal control diet (NCD, 3.85 kcal/g, D12450B, 10% kcal from fat, Research Diets); and 5) the NSJP group, mice were fed an NCD and treated with 2 g/kg of SJP. SJP or orlistat supplementation was provided daily by oral gavage for 8 weeks. Body weights were recorded weekly. After 8 weeks, all mice were fasted for 12 h, and blood samples were collected from the orbital vascular plexus. Fresh feces and tissues were immediately collected after the mice were euthanized by cervical dislocation.

### 2.6 Biochemical Assays and Histological Analysis

The serum levels of glucose, alanine aminotransferase (ALT), aspartate aminotransferase (AST), total cholesterol (TC), triglycerides (TG), low-density lipoprotein cholesterol (LDLC), and high-density lipoprotein cholesterol (HDLC) were measured in accordance with the methodology proposed in manuals that came along with the commercial assay kits from Nanjing Jiancheng Technology Co. (Nanjing, China). Serum leptin, adiponectin, and insulin levels were analyzed using ELISA kits (Shanghai Enzyme Linked Biotechnology Co., Ltd., Shanghai, China). Epididymal and subcutaneous adipose tissues were fixed in 4% paraformaldehyde, embedded in paraffin wax, sliced (5 µm), and stained using H&E. Images were obtained using an Olympus AX80 microscope (Olympus Optical, Tokyo, Japan) equipped with a Nikon D2X high-resolution camera (Nikon, Tokyo, Japan).

### 2.7 Short-Chain Fatty Acid Analysis of Fecal Samples

To measure SCFA content, fecal samples were homogenized with distilled water and acidified using 5 M of H_3_PO_4_ (González-Hernández et al., 2019). The supernatants were obtained after centrifugation at 10,000 × *g* for 5 min at 4°C and were analyzed as per the method described in Section 2.4.3.

### 2.8 Fecal DNA Extraction and 16S rRNA Amplicon Sequencing

Fecal DNA was extracted using the E. Z.N.A.^®^ soil DNA Kit (Omega Bio-Tek, Norcross, GA, USA) according to the manufacturer’s procedures. The V3–V4 region was amplified using universal primers (338F 5′-ACT​CCT​ACG​GGA​GGC​AGC​AG-3′ and 806R 5′-GGACTACHVGGGTWTCTAAT-3′). Paired-end sequencing libraries (2 × 300 bp) were sequenced on a MiSeq platform (Illumina, San Diego, USA), as per the procedures specified by Majorbio Bio-Pharm Technology Co., Ltd. (Shanghai, China). Raw reads were quality-filtered using Trimmomatic, merged by FLASH (version 1.2.11), and processed using QIIME (version 1.9.1). Operational taxonomic units were clustered at 97% similarity (UPARSE, version 7.0.1090). The taxonomy was assigned using the RDP Classifier (version 11.5, http://rdp.cme.msu.edu/) against the Bacterial SILVA database (0.7 confidence threshold, version 132). Bioinformatics analyses were performed on the Majorbio I-Sanger Cloud Platform (http://www.i-sanger.com). Beta diversity was estimated using principal coordinate analysis based on the Bray–Curtis dissimilarity. Analysis of similarities (ANOSIM) values were analyzed using the Vegan R Package. Significant differences among multiple groups at the phylum, family, and genus levels were analyzed using the Kruskal–Wallis test with false discovery rate (FDR) correction. Linear discriminant analysis (LDA) of effect size (LEfSe) of gut microbiota was analyzed at Log LDA >3.5.

### 2.9 DNA Extraction and Quantification of *Akkermansia muciniphila* Abundance in Single-Strain Analysis

*A. muciniphila* DSM22959 was cultured in modified peptone–yeast extract–glucose (PYG) medium (DSMZ Medium 104) supplemented with 0.25% porcine gastric mucin (type III; Sigma-Aldrich, St. Louis, MO, USA). To evaluate the effect of SJP on the growth of *A. muciniphila*, individual analysis groups were created whereby the glucose in the original PYG medium was replaced independently with 10 mg/ml of SJP, fucose, and Algo. A control group was prepared without the addition of any experimental compounds. Each group was cultured at 37°C in a YQX-T anaerobic incubator (Longyue Instrument Equipment Co., Ltd., Shanghai, China) in the presence of a mixture of H_2_ (10%), CO_2_ (10%), and N_2_ (80%). Each experimental group was evaluated in triplicate. Samples were collected after 48 h for spectrophotometric analysis at 600 nm (OD_600_) and DNA extraction. DNA was extracted using a TIANamp bacteria DNA kit (DP302; Tiangen Biotech Co., Ltd., Beijing, China), and the concentration of DNA was measured using a Nano-100 micro-spectrophotometer. RT-qPCR was performed using ABI QuantStudio3 (Thermo Fisher Scientific, Waltham, USA) and Standard curves were generated using the diluted DNA of *A. muciniphila* DSM22959. The following primer sequences were used: forward 5′-CAC​ACC​GCC​CGT​CAC​AT-3′ and reverse 5′-TGC​GGT​TGG​CTT​CAG​ATA​CTT-3′. And quantification was performed in triplicate, in accordance with a previous report (Roopchand et al., 2015).

### 2.10 Statistical Analysis

Data are presented as mean ± SD. During *in vitro* fecal treatment and single *A. muciniphila* cultivation, differences in pH and OD_600_ values were assessed by one-way ANOVA with post-hoc Tukey’s honestly significant difference (HSD) test. The Kruskal–Wallis with Games–Howell test was used to assess the differences in SCFA production. For animal experiments, weight gain, total energy intake, liver and epididymal fat weight, and serum parameters were assessed using the Kruskal–Wallis with Games–Howell test. SCFA production in feces was assessed using one-way ANOVA with post-hoc Tukey HSD test. Differences were considered significant when *p* < 0.05. Statistical analyses were performed using SPSS 22.0 software (SPSS, Inc., USA).

## 3 Results

### 3.1 General Analysis of Seaweed *Laminaria japonica* Polysaccharides

**FIGURE 1:**
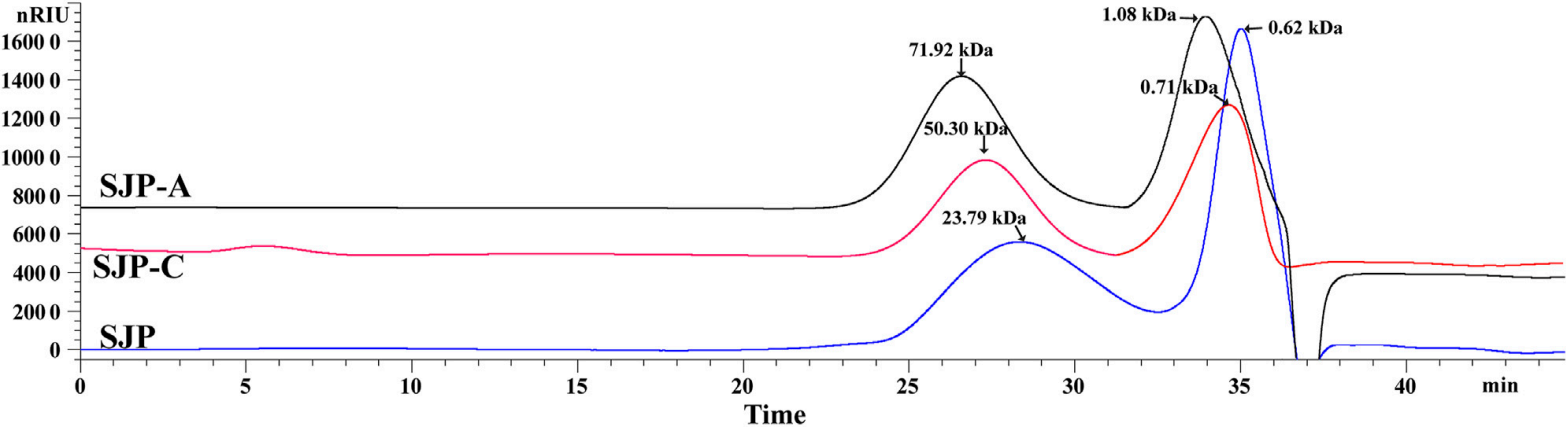
HPSEC profiles of SJP, SJP-A, and SJP-C. HPSEC, high-performance size exclusion chromatography; SJP, seaweed *Laminaria japonica* polysaccharides.

The extraction yields of low-MW SJP are shown in Table 1. The highest yield obtained was 41.83% in the composite enzymatic hydrolysis group (SJP), which was higher than using single cellulase (SJP-C) or alginate lyase 102C300C (SJP-A). SJP-C obtained a higher yield rate of 25.08% compared with SJP-A without significant difference. After pressure pretreatment and enzymatic hydrolysis, low-MW SJP with a major component of carbohydrates (more than 78%), and a small proportion of ash (6.49–7.02%) and protein (2.29–2.98%) were obtained (Table 1). Monosaccharide composition in SJP samples includes glucose (28.85%), mannuronic acid (20.45%), guluronic acid (12.27%), fucose (17.31%), galactose (9.82%), mannose (8.04%), and a small amount of glucuronic acid (3.26%). Higher proportions of mannuronic acid and guluronic acid were obtained using alginate lyase 102C300C, whereas hydrolysis using single cellulase enhanced the glucose content in the SJP-C group. No significant differences in sulfate contents were observed with varied enzymatic hydrolysis. However, SJP and SJP-A showed a higher uronic acid content than SJP-C (*p* < 0.05). Two main peaks were observed in high-performance size exclusion chromatography (HPSEC) of SJP-A samples with molar mass distribution averages of 71.92 and 1.08 kDa (Figure 1). A lower molar mass distribution of 50.30 and 0.71 kDa was shown in SJP-C. However, better degradation efficiency and lower molar mass distribution were obtained in SJP using both alginate lyase 102C300C and cellulase with MW averages of 23.79 and 0.62 kDa (Figure 1).

**TABLE 1:** Chemical characterization of SJP, SJP-A, and SJP-C.

Sample	Yield rate (%)	g/100 g				Monosaccharides (mol%)^*^							Uronic acid (%)	Sulfate (%)
		Protein	Ash	Carbohydrate	Moisture	Glc	ManA	GulA	Fuc	Gal	Man	GlcA		
SJP	41.83 ± 1.17^b^	2.29 ± 0.11^a^	6.49 ± 0.62^a^	80.14 ± 1.47^a^	4.48 ± 0.22^a^	28.85	20.45	12.27	17.31	9.82	8.04	3.26	41.19 ± 1.87^b^	9.85 ± 0.21^a^
SJP-A	23.21 ± 0.89^a^	2.58 ± 0.15^a^	7.02 ± 0.54^a^	78.93 ± 1.33^a^	4.01 ± 0.18^a^	26.31	18.32	14.79	18.31	10.09	7.98	4.20	40.21 ± 1.20^b^	10.21 ± 0.76^a^
SJP-C	25.08 ± 1.03^a^	2.98 ± 0.14^a^	6.54 ± 0.37^a^	79.37 ± 1.15^a^	4.19 ± 0.19^a^	36.15	15.32	8.79	16.45	9.09	8.94	5.26	30.21 ± 0.79^a^	8.93 ± 0.47^a^

Note. Results are shown as mean ± SD (*n* = 3).

Glc, glucose; ManA, mannuronic acid; GulA, guluronic acid; Fuc, fucose; Gal, galactose; Man, mannose; GlcA, glucuronic acid; HSD, honestly significant difference.

^*^The mole ratio (mol%) of each monosaccharide is calculated based on its content to the total measured monosaccharide content.

^a,b^ Significant differences were analyzed by one-way ANOVA, with post-hoc Tukey HSD, test for multiple groups comparisons (*p* < 0.05).

### 3.2 Seaweed *Laminaria japonica* Polysaccharide Utilization Profile During *In Vitro* Fecal Treatment

**FIGURE 2:**
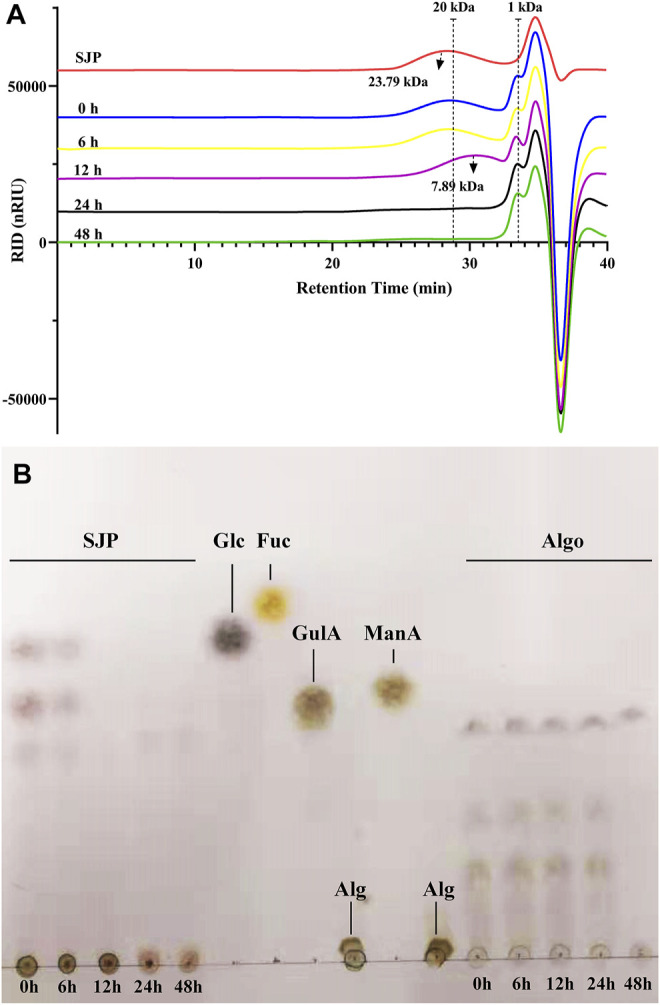
SJP utilization during *in vitro* human fecal treatment. **(A)** HPSEC profiles depicting SJP utilization. **(B)** TLC profiles depicting the degradation of oligosaccharides in SJP and Algo samples. The major monosaccharides in SJP including glucose (Glc), mannuronic acid (ManA), guluronic acid (GulA), and fucose (Fuc) and the origin alginate sample (Alg) were used for reference. SJP, seaweed *Laminaria japonica* polysaccharides; HPSEC, high-performance size exclusion chromatography; TLC, thin-layer chromatography.

To profile the utilization of SJP by intestinal bacteria, the supernatant following fecal treatment of the SJP was evaluated using HPSEC (Figure 2A). In the initial 6 h of the reaction, SJP were slowly utilized, and no obvious changes were observed. After 12 h, the SJP were rapidly utilized by fecal microbiota, and the average MW decreased from 23.79 to 7.89 kDa (Figure 2A). After 24 h, all high-MW fractions were completely degraded. More detailed utilization profiles of SJP were obtained by TLC (Figure 2B). The variations in the color densities of the spots in the TLC profile revealed the utilization of oligomer components in SJP and Algo by the human fecal microbiota (Figure 2B). The disappearance of upper spots within 12 h indicated that SJP was quickly consumed during *in vitro* fecal treatment; however, no obvious utilization was observed within 24 h of fecal treatment of Algo. The TLC graphs revealed that SJP contained considerable proportions of polymeric components around the origin spotting area. Obvious utilization was observed within 24 h of fecal treatment of polymeric components in SJP owing to the similar fading color.

### 3.3 pH Variation and Short-Chain Fatty Acid Production During *In Vitro* Fecal Treatment

**FIGURE 3:**
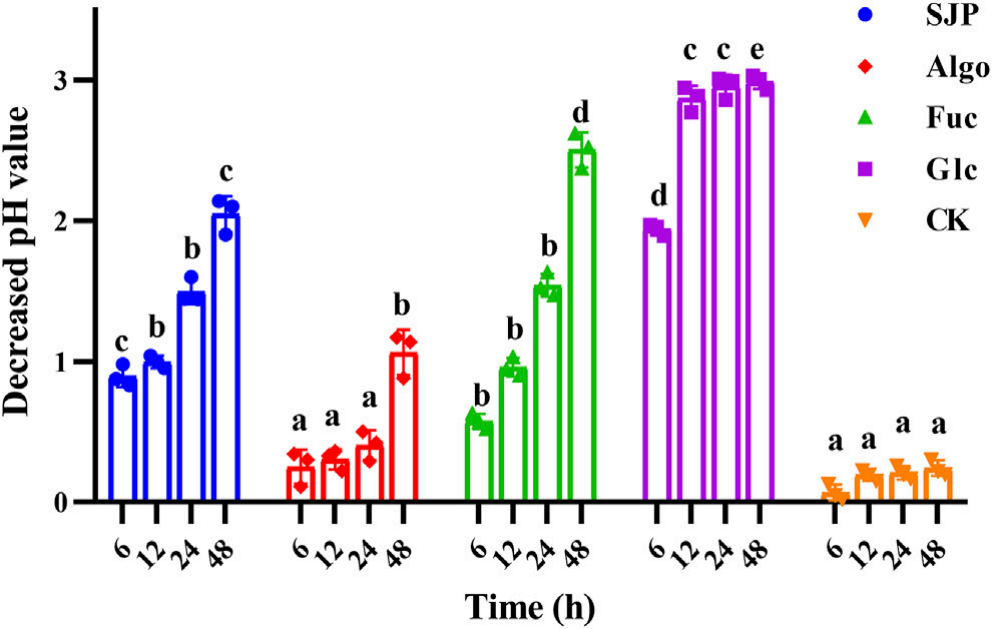
pH variation during *in vitro* degradation. The pH reduction during the *in vitro* degradation of SJP, alginate oligosaccharides (Algo), fucose (Fuc), and glucose (Glc). The control condition (CK) was achieved by excluding the addition of experimental substances. Results are shown as mean ± SD (*n* = 3). ^a,b,c,d^ Significant differences were analyzed by one-way ANOVA with post-hoc Tukey HSD test for multiple groups comparisons at the same fermentation time (*p* < 0.05). SJP, seaweed *Laminaria japonica* polysaccharides; HSD, honestly significant difference.

The change in pH during fecal treatment of SJP *in vitro* was monitored (Figure 3). Algo and fucose were used for comparison, as they are the main components of SJP. A slight reduction in pH was observed during the reaction in the control group conditions within 0–48 h. Glucose resulted in the highest reduction in pH (2.99 units) after 48 h, followed by fucose (2.50 units). The most rapidly decreasing pH value was observed with glucose within the first 6 h, followed by the SJP (0.90 units) and fucose (0.57 units). SJP were rapidly and consistently degraded into acid, resulting in a continuous decrease in pH throughout the course of the reaction. Algo was degraded slowly, and an overall reduction of 1.06 units was observed in the pH after 48 h.

**FIGURE 4:**
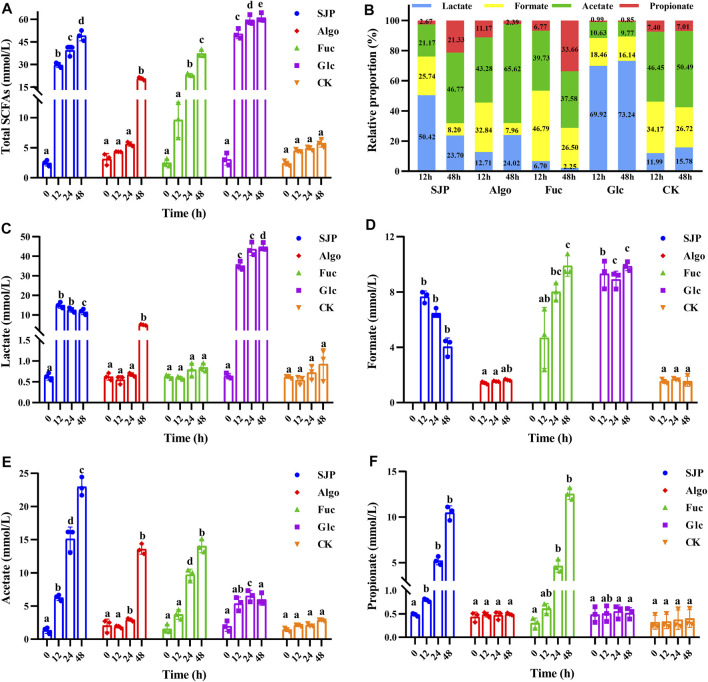
SCFA production during the *in vitro* degradation of SJP, alginate oligosaccharides (Algo), fucose (Fuc), and glucose (Glc). The control condition (CK) was achieved by excluding the addition of experimental substances. The concentrations of **(A)** total SCFAs, **(C)** lactate, **(D)** formate, **(E)** acetate, and **(F)** propionate were determined at 0, 12, 24, and 48 h, respectively. **(B)** Relative proportion of individual SCFAs at 12 and 48 h. Results are shown as mean ± SD (*n* = 3). ^a,b,c,d^ Significant differences were analyzed using Kruskal–Wallis with Games–Howell test for multiple groups comparisons at the same fermentation time (*p* < 0.05). SCFA, short-chain fatty acid; SJP, seaweed *Laminaria japonica* polysaccharides.

In this study, glucose showed the most rapid acid-producing activity, and the total SCFAs increased from 3.08 mM at 0 h to 50.38 mM at 12 h (Figure 4A). Considerable rapid SCFA-producing activity was also observed with SJP. The total SCFA concentration increased from 2.46 mM at 0 h to 29.78 mM with SJP at 12 h, which was significantly higher than that observed with fucose (9.62 ± 2.93 mM), the control (4.34 ± 0.03 mM), and Algo (4.50 ± 0.22 mM) conditions (*p* < 0.05). The highest SCFA concentration was observed in the glucose group (61.19 mM) after 48 h, followed by the SJP group (49.19 ± 3.40 mM). Both fucose and Algo were utilized slowly by human fecal microbiota, which was consistent with the pH values obtained (Figure 3). Algo utilization did not result in significant SCFA production until 48 h (37.31 ± 2.35 mM) in comparison with the control (5.75 ± 0.65 mM), which was consistent with the utilization rates determined using TLC (Figure 2B).

The high yield of SCFAs with glucose was mainly attributed to a higher production of lactate. The lactate concentration reached 35.20 and 44.81 mM at 12 and 48 h, respectively (Figure 4C, *p* < 0.05), accounting for 69.92% and 73.24% of the total SCFAs, at 12 and 48 h, respectively (Figure 4B). SJP utilization also resulted in considerable lactate production, reaching 15.04 mM at 12 h (*p* < 0.05). In contrast, lower lactate levels were observed with both fucose and Algo. Glucose degradation resulted in a high amount of formate (9.31 ± 1.02 mM) at 12 h, which reached 9.86 mM at 48 h, with a similar trend being observed with fucose (Figure 4D). However, SJP were observed to exhibit a reduced tendency to produce formate after 12 h. In this study, SJP utilization resulted in maximum acetate production as compared with other groups (*p* < 0.05), reaching 15.11 and 22.97 mM at 24 and 48 h, respectively (Figure 4E). Moreover, the utilization of fucose and Algo also resulted in considerable acetate production, with the concentrations reaching 14.02 and 13.57 mM at 48 h, respectively (Figure 4E). During the entire process, significant propionate concentration was only observed with SJP and fucose (*p* < 0.05, Figure 4F), and no butyrate was detected.

### 3.4 Effects of Seaweed *Laminaria japonica* Polysaccharides on Preventing High-Fat Diet-Induced Obesity in Mice

**FIGURE 5:**
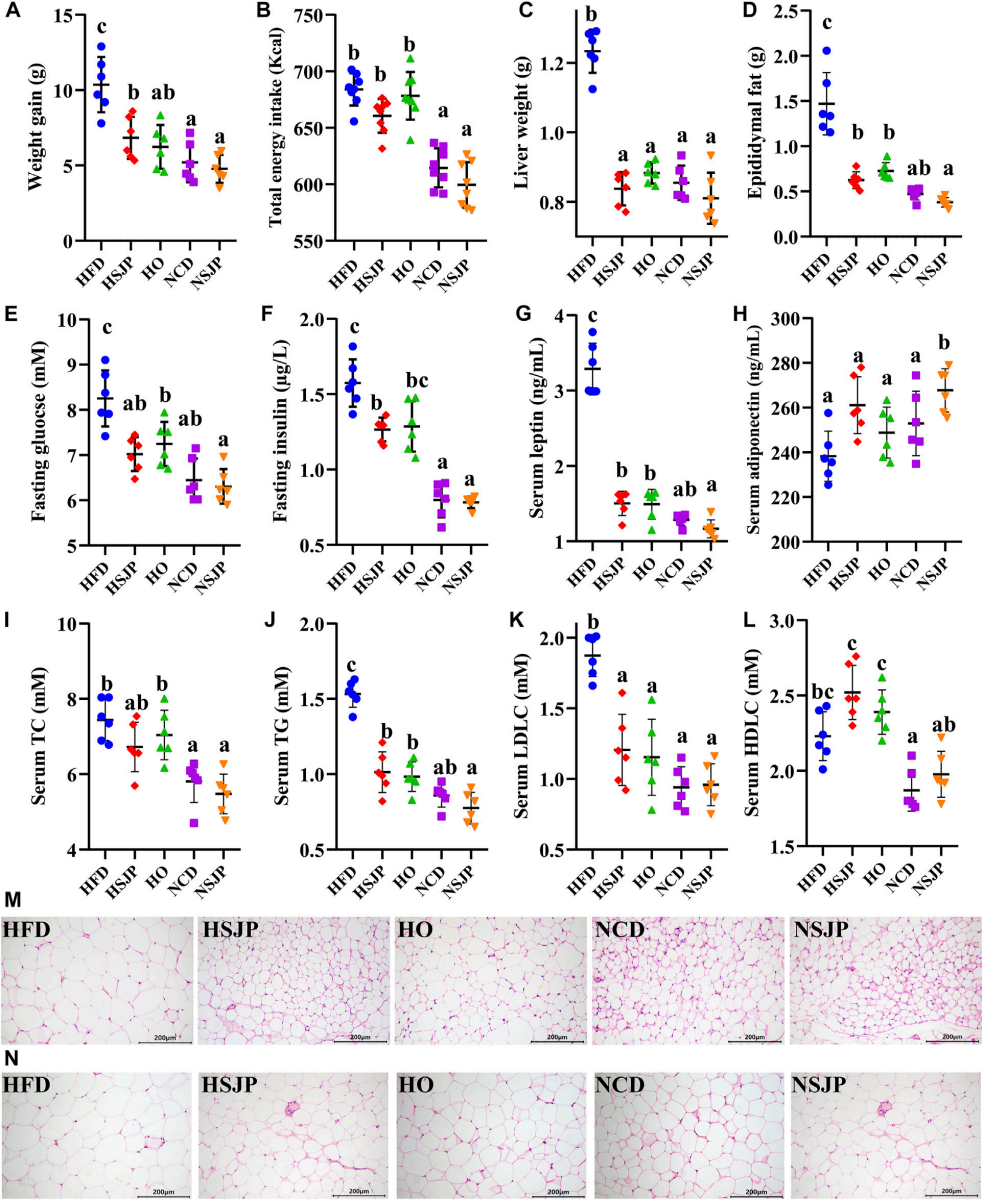
Effects of SJP with respect to preventing HFD-induced weight accumulation and associated metabolic syndromes in mice after 8 weeks of supplementation. **(A)** Weight accumulation, **(B)** total daily energy intake in each group, **(C)** liver weight, **(D)** epididymal fat weight, **(E)** level of fasting glucose, **(F)** fasting serum insulin, **(G)** leptin, **(H)** adiponectin, **(I)** TC, **(J)** TG, **(K)** LDLC, and **(L)** HDLC in serum. Representative H&E-stained images of **(M)** epididymal and **(N)** subcutaneous adipocyte (scale bar, 200 mm). Results are shown as mean ± SD (*n* = 6). ^a,b,c^ Significant differences were analyzed using Kruskal–Wallis with Games–Howell test (*p* < 0.05). SJP, seaweed *Laminaria japonica* polysaccharides; HFD, high-fat diet; TC, total cholesterol; TG, triglycerides; LDLC, low-density lipoprotein cholesterol; HDLC, high-density lipoprotein cholesterol.

SJP supplementation significantly reduced body weight gain (Figure 5A, *p* < 0.05), liver weight (Figure 5C, *p* < 0.05), and epididymal adipose tissue weight (Figure 5D, *p* < 0.05) in HFD-fed mice. However, no significant differences in daily energy intake were observed among all the HFD-fed or NCD-fed groups (Figure 5B). SJP supplementation reduced the fasting glucose (Figure 5E, *p* < 0.05) and fasting insulin (Figure 5F, *p* < 0.05) levels and ameliorated serum hyperlipidemia with a reduction in the levels of TC (Figure 5I, *p* < 0.05), TG (Figure 5J, *p* < 0.05), and LDLC (Figure 5K, *p* < 0.05) in HFD-fed mice. Moreover, leptin concentration was reduced (Figure 5G, *p* < 0.05) in the HSJP group, in comparison with that in the HFD group, while the serum adiponectin (Figure 5H) and HDLC (Figure 5L) levels increased in response to SJP supplementation. Additionally, SJP supplementation significantly decreased fat deposition in epididymal and subcutaneous tissues and reduced the adipocyte size (Figures 5M,N). The HO group exhibited reduced weight gain (Figure 5A), TG (Figure 5J), and LDLC (Figure 5K) levels, compared with the HSJP group, while exhibiting slightly higher liver weight (Figure 5C), and fasting glucose (Figure 5E), fasting insulin (Figure 5F), and serum TC (Figure 5I) levels. Moreover, SJP supplementation exhibited the potential to improve serum glucose and lipid contents in mice fed an NCD. Serum adiponectin levels were significantly increased in the NSJP group, in comparison with those in the NCD group (Figure 5H, *p* < 0.05). The same trend was observed for epididymal adipose tissue weight (Figure 5D) and adipocyte size (Figures 5M,N) in the NSJP group.

### 3.5 Fecal Short-Chain Fatty Acid Concentrations

In comparison with the NCD group, the HFD group exhibited decreased SCFA concentration in feces (*p* < 0.05, Table 2). Supplementation with SJP increased fecal total SCFAs in HFD- and NCD-fed mice (*p* < 0.05). SJP supplementation also increased the lactate, acetate (*p* < 0.05), and butyrate levels in the fecal samples of HFD-fed mice. Higher concentrations of butyrate (*p* < 0.05) were observed in the HO group; however, a change in total fecal SCFAs was not evident in the HO group. Supplementation with SJP increased the levels of acetate (*p* < 0.05) in the NCD group. Moreover, in comparison with the NCD group, a trend of increasing lactate, propionate, and butyrate levels was observed in the NSJP group.

**TABLE 2:** Fecal SCFAs in HFD-fed C57BL/6 mice supplemented with SJP.

Fecal SCFAs, μmol/g	HFD	HSJP	HO	NCD	NSJP
Lactate	2.47 ± 0.07^a^	4.08 ± 0.11^ab^	2.89 ± 0.05^a^	6.18 ± 0.13^c^	8.05 ± 0.19^c^
Acetate	23.45 ± 1.56^a^	26.43 ± 1.09^b^	21.23 ± 1.38^a^	30.20 ± 1.53^c^	33.93 ± 1.28^d^
Propionate	0.97 ± 0.10^a^	1.47 ± 0.09^a^	0.78 ± 0.04^a^	1.99 ± 0.10^ab^	2.57 ± 0.05^b^
Iso-butyrate	0.13 ± 0.05^a^	0.12 ± 0.03^a^	0.20 ± 0.01^a^	0.26 ± 0.01^ab^	0.29 ± 0.02^b^
*n*-Butyrate	0.24 ± 0.02^a^	0.56 ± 0.05^ab^	0.79 ± 0.04^b^	1.98 ± 0.05^c^	2.45 ± 0.07^c^
*n*-Valerate	0.30 ± 0.02^a^	0.44 ± 0.08^a^	0.23 ± 0.01^a^	0.43 ± 0.05^a^	0.39 ± 0.02^a^
Total SCFAs	27.56 ± 1.18^a^	33.10 ± 1.67^b^	26.12 ± 1.82^a^	41.04 ± 1.93^c^	47.68 ± 2.16^d^

Note. Results are shown as mean ± SD (*n* = 6).

SCFA, short-chain fatty acid; HFD, high-fat diet; SJP, seaweed *Laminaria japonica* polysaccharides; HSD, honestly significant difference.

^a,b,c,d^ Significant differences were analyzed by one-way ANOVA, with post-hoc Tukey HSD, test for multiple groups comparisons (*p* < 0.05).

### 3.6 Response of Microbiota to Supplementation With Seaweed *Laminaria japonica* Polysaccharides in C57BL/6 Mice

**FIGURE 6:**
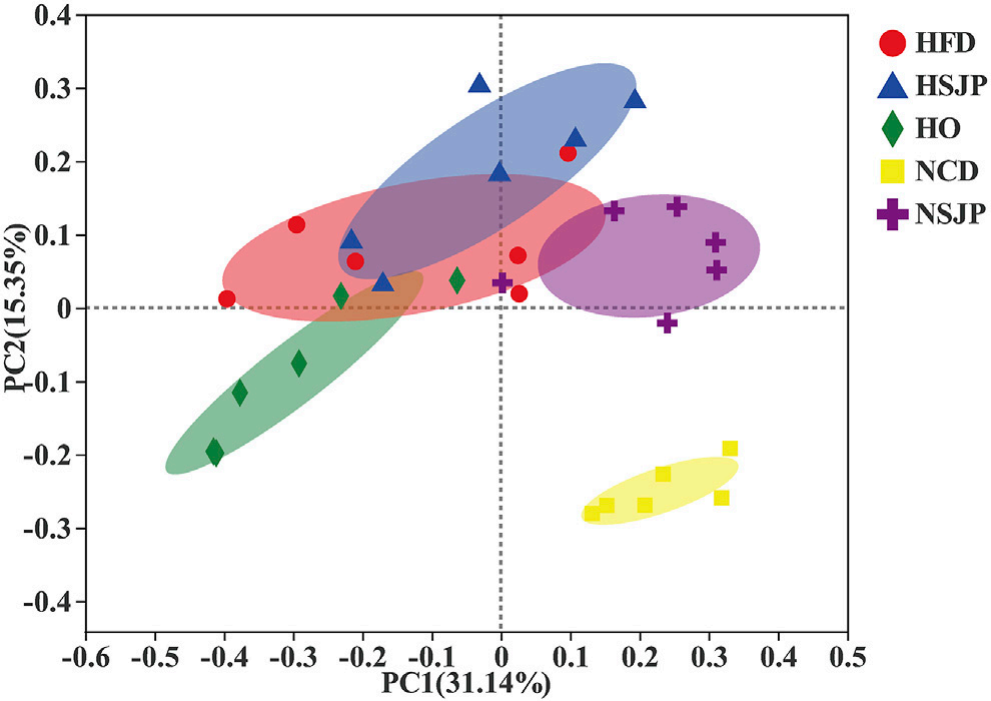
Microbiota response to dietary supplementation with SJP in C57BL/6 mice. Principal coordinate analysis plot of microbiota in C57BL/6 mice with SJP supplementation, based on the Bray–Curtis distance. SJP, seaweed *Laminaria japonica* polysaccharides.

The *in vivo* effects of SJP on intestinal microbiota composition were assessed *via* high-throughput sequencing of bacterial 16S rDNA in the feces. All groups were segregated by PC1 filtering and distinct clustering of their microbiota composition, which cumulatively accounted for 31.14% of the total variation (Figure 6). ANOSIM revealed that r = 0.65, *p* = 0.001. An intense shift was observed between the NCD and HFD groups, which indicates that HFD has a pronounced effect on the composition of fecal microbiota. Supplementation with SJP in both the HSJP and NSJP groups resulted in clear differences, in comparison with the control group, indicating that SJP affect the structure of the microbiota. Similarly, clear differences in bacterial compositions were observed between the HO and HFD groups.

**FIGURE 7:**
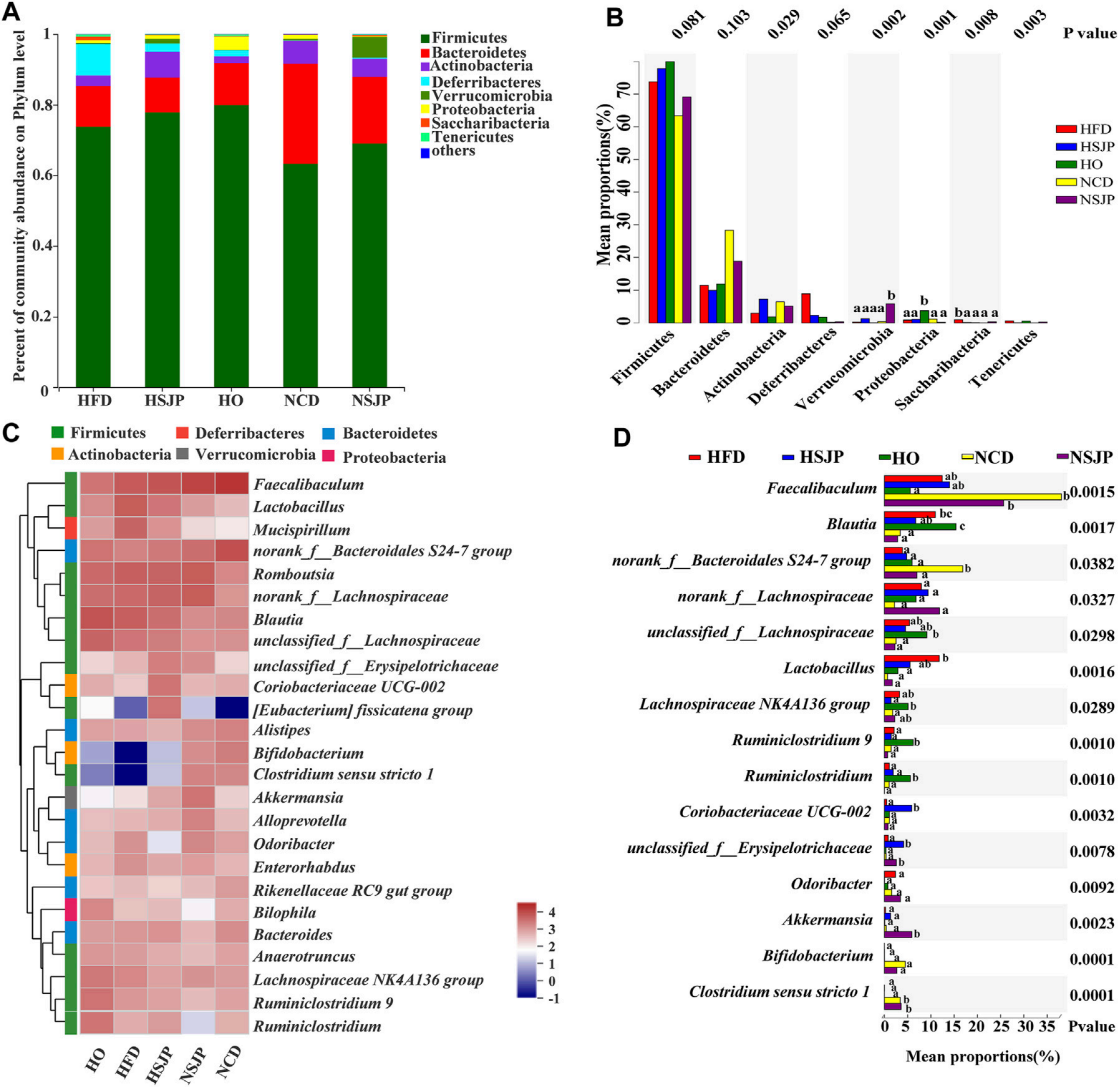
Bacterial taxonomic structures of gut microbiota at the phylum and genus levels in different C57BL/6 mice groups. **(A)** Relative abundance of gut microbes at the phylum level. The *y*-axis presents the mean relative abundance. **(C)** Heat map of the top 25 genera. ^a,b,c^ Significant differences among multiple groups at the **(B)** phylum and **(D)** genus levels were analyzed using Kruskal–Wallis tests with FDR correction (*p* < 0.05). N = 6 for each group. SJP, seaweed *Laminaria japonica* polysaccharides; FDR, false discovery rate.

At the phylum level, the intestinal microbiota is mainly composed of Firmicutes, Bacteroidetes, Actinobacteria, Deferribacteres, Verrucomicrobia, and Proteobacteria (Figure 7A). In both the HFD and NCD groups, SJP supplementation increased the abundance of Firmicutes, but the abundance of Bacteroidetes did not change significantly. A significantly higher abundance of Saccharibacteria was observed in all HFD groups, compared with that in all other groups (*p* < 0.05, Figure 7B), while Verrucomicrobia and Proteobacteria were the most abundant in the NSJP and HO groups, respectively (*p* < 0.05, Figure 7B). Besides, the abundance of Deferribacteres was increased in the HFD groups compared with that in the NCD groups. However, the abundance of Deferribacteres decreased upon treatment with SJP as well as orlistat.

Microbiota taxon shifts at the genus level are shown in Figures 7C and 7D. HFD reduced the abundance of *Faecalibaculum*, *Bifidobacterium*, *Bacteroidales S24-7 group*, and *Clostridium sensu stricto 1*, while increasing the abundance of *Blautia*, *Lactobacillus*, *Odoribacter*, *Mucispirillum*, and Lachnospiraceae *NK4A136 group*, compared with that observed in the NCD group (Figure 7C). HFD decreased the abundance of *Faecalibaculum* significantly, with the most pronounced decrease being observed in the HO group (*p* < 0.05, Figure 7D). Besides, a decreased abundance in *Bifidobacterium* was found in the HFD group. However, neither SJP nor orlistat treatment resulted in a significant increase in the abundance of *Bifidobacterium* (Figure 7C). The abundance of butyrate-producing bacteria, *Bacteroidales S24-7 group* and *Clostridium sensu stricto 1*, was decreased in the HFD group compared with the NCD group (*p* < 0.05, Figure 7D). However, SJP promoted the abundance of *Clostridium sensu stricto 1*, which was positively correlated with an increase in butyrate levels in the fecal and cecal samples (Table 2). Moreover, SJP enhanced the abundance of *Romboutsia* in the HSJP and NSJP groups (Figure 7C), which may be attributed to the high percentage of fucose in SJP (Table 1).

LEfSe was performed to identify the specific biomarkers that can account for differences between the HFD, NCD, HSJP, NSJP, and HO groups (Supplementary Figure S2). Our results confirmed the significant enrichment of *Blautia* and *Lactobacillus* in the HFD group. The potentially pathogenic phylotypes Moraxellaceae—and its representative genus *Acinetobacter*—were enriched in the HFD group (Supplementary Figure S2A). Additionally, we conclude that Saccharibacteria can likely account for the differences observed between the HFD group and the HSJP and HO groups (Supplementary Figures S2B,D). As shown in Supplementary Figures S2B,C, Verrucomicrobia—and its representative genus *Akkermansia*—were specifically enriched in the HSJP and NSJP groups. SJP supplementation increased the relative abundance of *A. muciniphila* by approximately 5.7- and 14.8-fold compared with that observed in the HFD and NCD control groups, respectively (Supplementary Figure S3).

### 3.7 Seaweed *Laminaria japonica* Polysaccharides Promote the Growth of *Akkermansia muciniphila*

**FIGURE 8:**
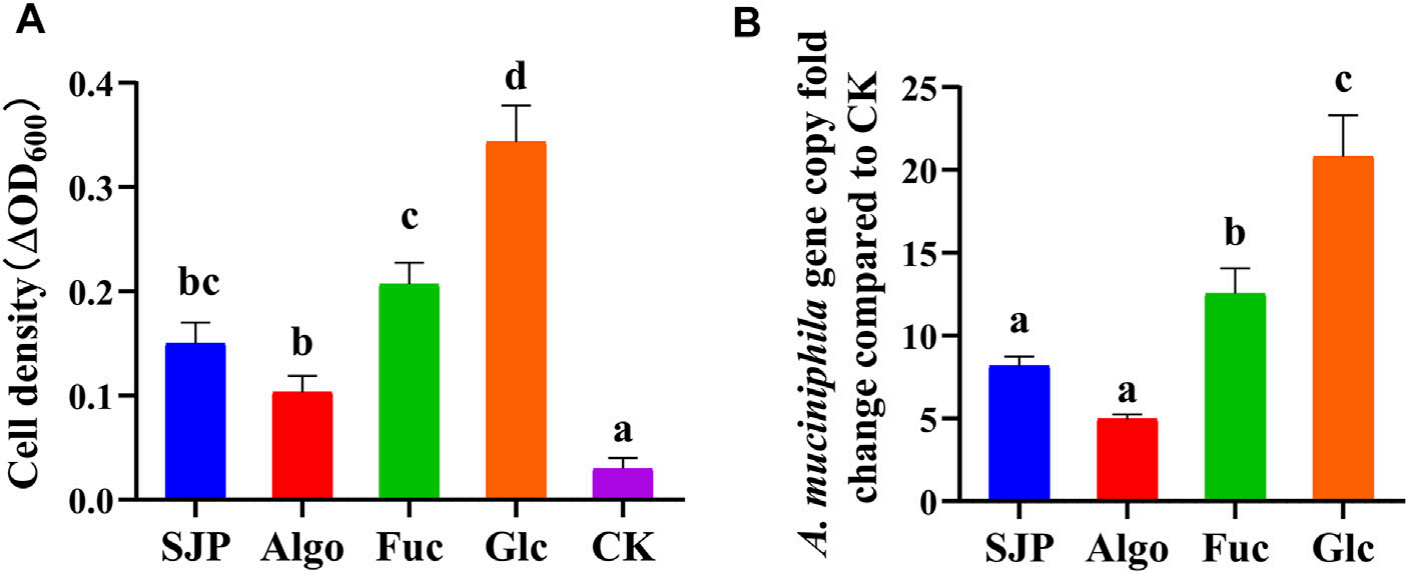
Growth of *Akkermansia muciniphila* in modified PYG medium in the presence of SJP, alginate oligosaccharides (Algo), fucose (Fuc), and glucose (Glc). The control condition (CK) was achieved by excluding the addition of experimental substances. **(A)** Changes in OD_600_ in each culture group. **(B)** Fold change in the *A. muciniphila* gene copy in each group relative to CK. Results are shown as mean ± SD (*n* = 3). ^a,b,c,d^ Significant differences were analyzed using one-way ANOVA with post-hoc Tukey HSD test for multiple group comparisons (*p* < 0.05). PYG, peptone–yeast extract–glucose; SJP, seaweed *Laminaria japonica* polysaccharides; HSD, honestly significant difference.

In this study, *A. muciniphila* was able to grow on PYG medium, without added sugar or polysaccharides; however, the OD_600_ increased by only 0.03, after 48 h (Figure 8A). SJP and Algo were found to promote the growth of *A. muciniphila* (OD_600_ values reached 0.15 and 0.10 after 48 h, respectively). Simple sugars, such as glucose and fucose, were more readily utilized as a food source, which resulted in OD_600_ values increasing to 0.34 and 0.21 after 48 h, respectively. A similar trend was observed upon the quantification of *A. muciniphila* by qPCR (Figure 8B). The *A. muciniphila* gene copy was 20.80-fold and 12.54-fold higher in the glucose and fucose groups, respectively, in comparison with the control group, followed by the SJP (8.17-fold) and Algo (4.96-fold) groups.

## 4 Discussion

Polysaccharides extracted from *L. japonica* (e.g., laminarin, alginate, and fucoidan) are regarded to have various functional properties, based on their role in regulating the intestinal microbiota and other beneficial physiological functions. However, they are quite difficult to obtain due to their considerable high viscosity and the difficulty associated with the degradation of alginate. The low extraction efficiency is a limiting factor for industrial use, largely due to the algae cell wall, which consists of complex polymers (Li et al., 2021). Therefore, enzyme-assisted methods can be considered as an effective technique to enhance the extraction efficiency (Wang et al., 2019). The MW is a critical factor, which influences the physiological functions of polysaccharides. Previous studies indicate that low-MW seaweed polysaccharides exhibited stronger biological activity than that of the initial polysaccharide (Zhou et al., 2012; Zha et al., 2016); however, more *in vivo* work should be carried out to investigate the relationship between MW of polysaccharides and their physiological functions. In this study, we developed a novel technology using combined high-pressure pretreatment (0.2 MPa for 10 min) and enzymatic degradation (150 U/g of recombinant alginate lyase and 200 U/g of cellulase) to extract low-MW polysaccharides from *L. japonica*. The extraction yields obtained 41.83% in the composite enzymatic hydrolysis group, which was higher than using single cellulase or alginate lyase 102C300C. Moreover, a lower molar mass distribution of polysaccharides was obtained in SJP compared with SJP-A and SJP-C. There was no significant difference in the uronic acid content between the SJP and SJP-A groups. However, SJP and SJP-A showed a higher uronic acid content as compared with SJP-C (*p* < 0.05). Alginate usually exists in the cell wall of brown algae in the form of macromolecules, which is combined with cellulose and other components. The MW of alginate in the cell wall of brown algae can be effectively reduced by alginate lyase treatment so that alginate oligosaccharides can be dissolved and released into the aqueous extract.

In recent decades, various monosaccharide constituents and chemical structures have been reported in polysaccharides isolated from *L. japonica*, which are related to different functional properties (Cui et al., 2016). Not only the MW but also the uronic acid contents are apparently the influential factors of the biological activity of seaweed polysaccharides (Zhao et al., 2008). Enzymatic methods using alginate lyase 102C300C in this work induced a higher proportion of mannuronic acid and guluronic acid in both the SJP and SJP-A groups, whereas a higher content of glucose was observed using single cellulase. Uronic acid-containing polysaccharides are known to offer unique physicochemical properties and bioactivities (Abdallah et al., 2020).

Seaweed polysaccharides have been reported to ameliorate metabolic and inflammatory diseases, including diabetes and obesity (Kasubuchi et al., 2015). Their fermentation products, including SCFAs, play an important role in providing energy to colonocytes, promoting bile acid excretion, and improving glucose tolerance (Koh et al., 2016; Alexander et al., 2019). Carbohydrate fermentation commonly occurs in the proximal colon, resulting in the accumulation of SCFAs and acidification of the intestinal environment (Govers et al., 1999). The change in pH during human fecal treatment of SJP *in vitro* was monitored compared with Algo and fucose, which are the main components of SJP. SJP showed more rapid and consistent acid-producing activity in comparison with Algo; further, this activity was confirmed *in vivo*. SCFAs are the major end products of gut microbiota-mediated polysaccharide fermentation. In this study, supplementation with SJP resulted in a marked increase in the acetate and lactate levels in both *in vitro* human fecal treatment and feces of HFD-fed mice. Acetate also functions as a secondary messenger to activate signaling pathways that increase the expression of the anorexigenic hormone leptin, reduce cholesterol synthesis, and stimulate GLP-1 and peptide YY release (Den Besten et al., 2013). SJP are valuable in the development of postbiotics to improve intestinal health in accordance with the decrease in weight gain in HFD-fed mice.

The regulating effect of gut microbiota on obesity has been confirmed using germ-free mice and fecal bacteria transplantation, and the manipulation of the gut microbiota provides a promising way for the improvement of obesity (Turnbaugh et al., 2006). In comparison with the NCD groups, HFD groups exhibited a higher relative abundance of Firmicutes but decreased relative abundance of Bacteroidetes, which is similar to the results obtained in a previous study (Lin et al., 2016). However, this trend is inconsistent with other reports, in which no significant difference in the abundance of Bacteroidetes and Firmicutes between obese and leaner individuals was observed (Zhao, 2013; Hae-Jin et al., 2015). Increased abundance of Deferribacteres has been previously reported in the gut microbiota of mice with HFD-induced obesity and dextran sodium sulfate-induced colitis (Berry et al., 2012; Serino et al., 2012). However, a decreasing abundance of Deferribacteres was observed upon treatment with SJP. Besides, SJP enhanced the abundance of *Romboutsia—*represented by producing bile salt hydrolase and urease enzymes and utilizes monosaccharides of l-fucose and sialic acid to produce formate, acetate, and lactate in both the HSJP and NSJP groups, which may be attributed to the high proportion of fucose in SJP (Gerritsen et al., 2017).

Marine seaweeds are recognized as a promising source of anti-obesity therapeutics. Bioactive extracts, including alginates, fucoidans, and fucoxanthin, have been extensively studied for their anti-obesity activity. Supplement of seaweed-extracted polysaccharides promoted the enhancement of beneficial bacteria including *Bifidobacterium*, *Lactobacillus*, or *Akkermansia* (Shang et al., 2018). Algo (degree of polymerization 1–4), fucoidans (from *L. japonica* and *Ascophyllum nodosum*), and fucoxanthin (from *Undaria pinnatifida*) have been reported to ameliorate HFD-induced metabolic syndromes, and this phenomenon was supposed to be associated with the increased abundance of *A. muciniphila* (Shang et al., 2017; Guo et al., 2019; Wang et al., 2020). *Akkermansia*, a Gram-negative bacterium belonging to Verrucomicrobia, has shown the potential as an emerging probiotic. Decreased abundance of *A. muciniphila* has been observed in mice affected by obesity or type 2 diabetes in a previous study, and oral *A. muciniphila* supplementation has been demonstrated to restore the thickness of mucus, enhance the gut barrier function, and reverse HFD-induced metabolic disorders (Everard et al., 2013; Xu et al., 2020). Amuc_1100, the nowadays isolated outer membrane of *A. muciniphila*, helps to explain the potential mechanism of the capacity of improving the gut barrier (Plovier et al., 2017). In this work, both the high-throughput sequencing of bacterial 16S rDNA in the feces and single *A. muciniphila* strain culture indicates that SJP contain considerable amounts of monosaccharides, including glucose, mannuronic acid, guluronic acid, and fucose, which may be utilized for the growth of *A. muciniphila*, and can be potentially used as therapeutic agents for HFD-induced obesity. However, the relationship between the anti-obesity effect of seaweed-extracted polysaccharides and the variation of *Akkermansia* has not been revealed clearly, and mechanism researches are further needed.

## 5 Conclusion

In conclusion, two issues of concern were addressed in this study. First, we developed a method for the efficient preparation of water-soluble polysaccharides from seaweeds. Owing to the high MW and viscosity of seaweed polysaccharides, it is difficult to achieve effective degradation and extraction using conventional techniques. In this study, a novel technology, combining high-pressure pretreatment (0.2 MPa for 10 min) and composite enzymatic degradation (150 U/g of recombinant alginate lyase and 200 U/g of cellulase), was established for preparing low-MW SJP. Second, seaweed is generally accepted as a healthy food by consumers; however, the mechanism by which seaweed aids in weight loss by modulating intestinal microorganisms is still unclear. In this study, we relied on an *in vitro* degradation system and HFD-fed mice model to prove that seaweed polysaccharides have a significant effect on weight loss and that this is achieved through improving the intestinal microbiota and increasing the synthesis of SCFAs. Therefore, this study not only provides a new technology suitable for the industrial development of seaweed polysaccharides but also provides insight into the mechanism by which seaweed polysaccharides promote weight loss through the improvement of the gut microbiota. Future research will focus on the metabolic mechanism of specific intestinal microbiota associated with the dietary supplementation of SJP, with the aim of increasing our understanding of this natural therapeutic and promoting its development for the treatment of obesity-related diseases.

## Data Availability

The original contributions presented in the study are included in the article/Supplementary Material, further inquiries can be directed to the corresponding authors. The 16S rRNA gene sequences presented in the study are deposited in the NCBI SRA (Short Read Archive) database, accession number PRJNA788439.

## Glossary

Algo: alginate oligosaccharides
ALT: alanine aminotransferase
ANOSIM: analysis of similarities
AST: aspartate aminotransferase
CK: control condition
Fuc: fucose
Gal: galactose
Glc: glucose
GlcA: glucuronic acid
GulA: guluronic acid
HDLC: high-density lipoprotein cholesterol
H&E: hematoxylin and eosin
HFD: high-fat diet
HO: HFD supplemented with orlistat
HPLC: high-performance liquid chromatography
HPSEC: high-performance size exclusion chromatography
HSJP: HFD supplemented with SJP
LDLC: low-density lipoprotein cholesterol
LEfSe: linear discriminant analysis of effect size
ManA: mannuronic acid
Man: mannose
MW: molecular weight
NCD: normal control diet
NSJP: NCD supplemented with SJP
PYG: peptone-yeast extract-glucose
TC: total cholesterol
TG: triglycerides
TLC: thin-layer chromatography
SCFAs: short-chain fatty acids
SJP: seaweed *L. japonica* polysaccharides
