# Supplementary material for: Enzymatic Preparation of Low-Molecular-Weight Laminaria japonica Polysaccharides and Evaluation of Its Effect on Modulating Intestinal Microbiota in High-Fat-Diet-Fed Mice
Source: Front Bioeng Biotechnol. 2022 Feb 14;9:820892. doi: 10.3389/fbioe.2021.820892 (PMC8883051; doi:10.3389/fbioe.2021.820892)
Supplement: Supplementary file 1 [file DataSheet1.DOC]

Supplementary Material


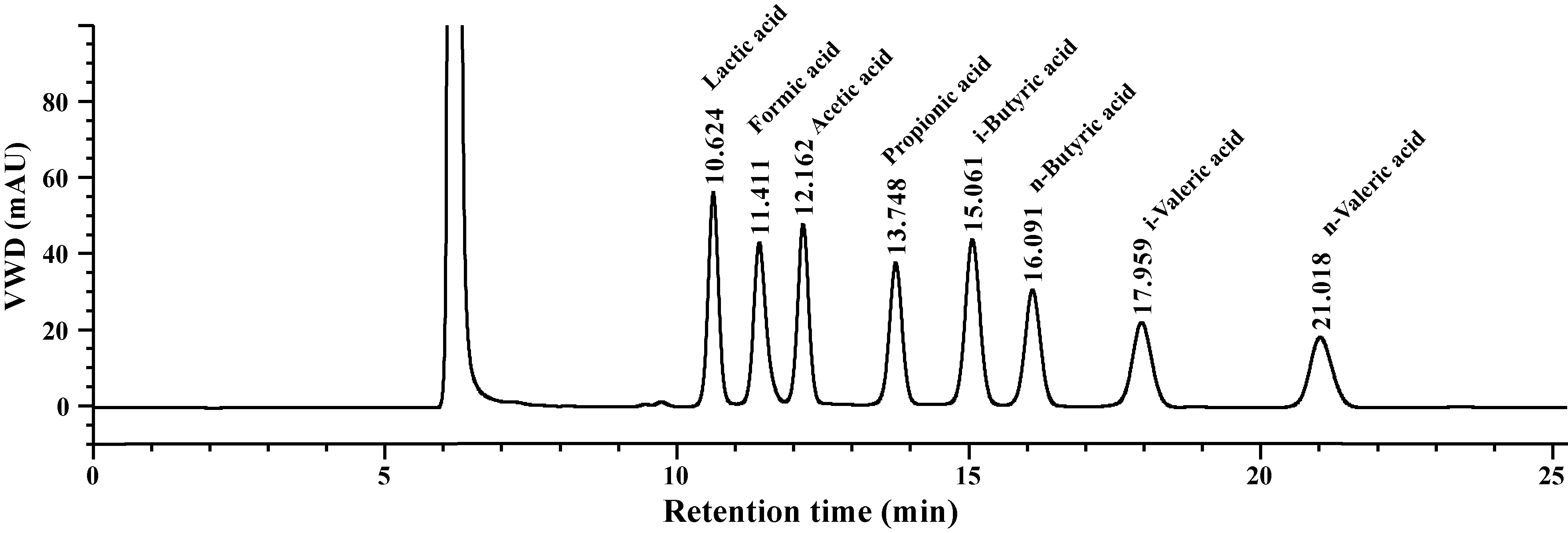


**Supplementary Figure S1** Chromatogram of experimental SCFAs standards. Lactic acid (10.96 mmol/L), formic acid (15.83 mmol/L), acetic acid (17.25 mmol/L), propionic acid (13.24 mmol/L), i-butyric acid (10.65 mmol/L), n-butyric acid (10.76 mmol/L), and i-valeric acid (8.94 mmol/L) were used as experimental standards. The n-valeric acid (9.08 mmol/L) was used as an internal standard.


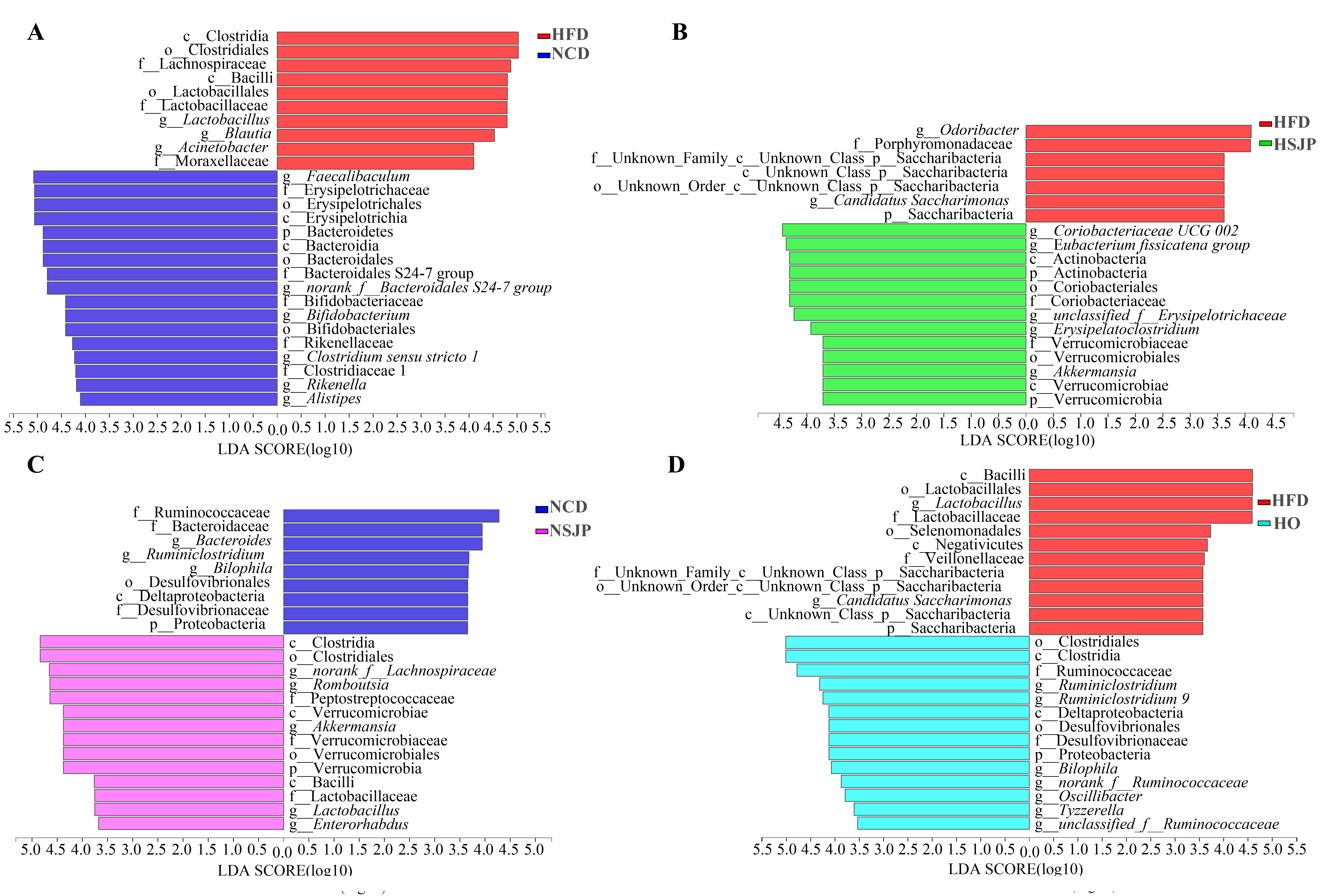


**Supplementary Figure S2** Linear discriminant analysis of effect size of gut microbiota in different C57BL/6 mice groups. Comparisons of (A) HFD group and NCD group, (B) HFD group and HSJP group, (C) NCD group and NSJP group, and (D) HFD group and HO group were analyzed to determine the taxa that best characterize each biological class (Log LDA > 3.5).





**Supplementary Figure S3** Proportion abundance of *Akkermansia* in different C57BL/6 mice groups. ^a.b^ Significant differences among multiple groups were analyzed by Kruskal-Wallis tests with FDR correction (*p* < 0.05).
